# 1386. Chagas Disease in Chile: an Old and Forgotten

**DOI:** 10.1093/ofid/ofad500.1223

**Published:** 2023-11-27

**Authors:** Catalina Novoa, Carolina Galindo, Camila Jorquera, María Laura Otth, Marcelo Wolff, Sandra Gonzalez

**Affiliations:** Hospital Clínico San Borja Arriarán, santiago, Region Metropolitana, Chile; Hospital Clínico San Borja Arriarán, santiago, Region Metropolitana, Chile; Hospital Clínico San Borja Arriarán, santiago, Region Metropolitana, Chile; Hospital Clínico San Borja Arriarán, santiago, Region Metropolitana, Chile; FUNDACION ARRIARAN, santiago, Region Metropolitana, Chile; Hospital Clínico San Borja Arriarán, santiago, Region Metropolitana, Chile

## Abstract

**Background:**

Chagas disease (ChD) affects 8-10 million people worldwide.

In Chile there are 98,000 people affected. Most endemic region is the middle northern part of the country, but not present south of the central areas. Significant interruption of vector transmission was achieved in the late 1900s. Since 2008, blood products for transfusions are screened universally, and since 2014 ChD serology testing has been implemented in all pregnant women, focusing efforts on prevention of vertical transmision, the most important form of contagion at present.

Being a referal center, we describe the demographic and clinical presentation as well as the treatment offered to the ChD referred patients.

**Methods:**

Descriptive, retrospective study of patients with ChD referred to the San Borja Arriarán Hospital between january 2009 and march 2023. Demographic and clinical variables were analyzed: likely transmission mechanism, reason for testing, classification up on admission, diagnostic laboratory tests, treatment and reported adverse drug reaction (ADRs)

**Results:**

*T. cruzi* infection was diagnosed in 234 patients, 74% women. The median age in years was 47; 70% Chileans nationals, the majority living in the Metropolitan Region (Fig 1). Among foreigners, the majority were form Bolivian. 76% had no associated comorbidity. Blood donation was the main form of detection (42 %), followed by pregnancy and family history study (Fig 2). Vector transmission was estimated in 50% of the cases; 84% were classified on admission as an indeterminate chronic disease; 42% patients received treatment, all with nifurtimox; 60% of them presented ADRs, more frequently during the first two weeks of treatment initiation. Most ADRs were gastrointestinal (anorexia, nausea and vomiting) (Fig 3); treatment was discontinued 20% of patients. Age and sex were not associated to higher rate of ADRs.

**Figure:**
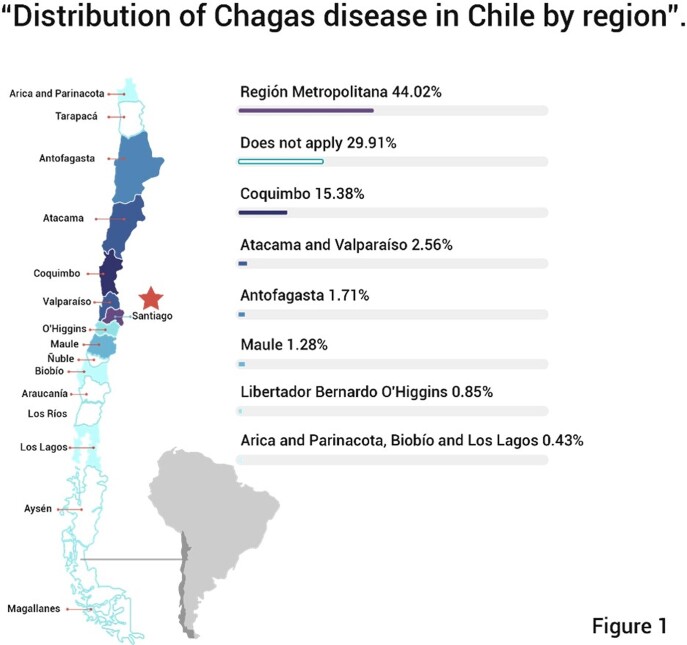

**Figure:**
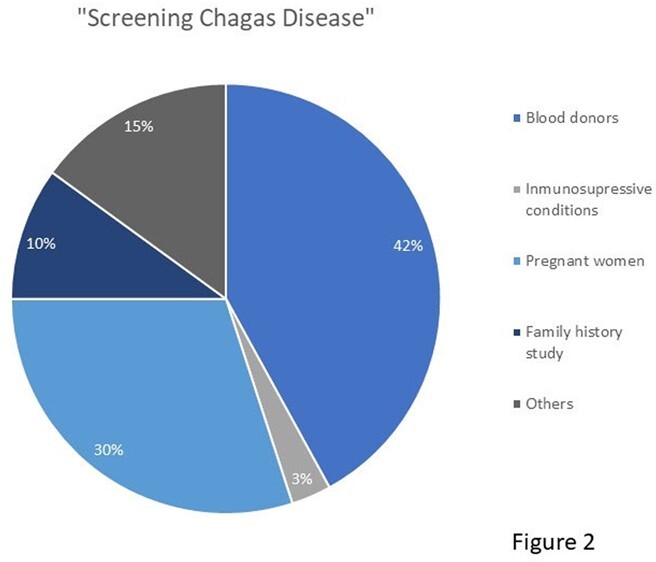

**Figure:**
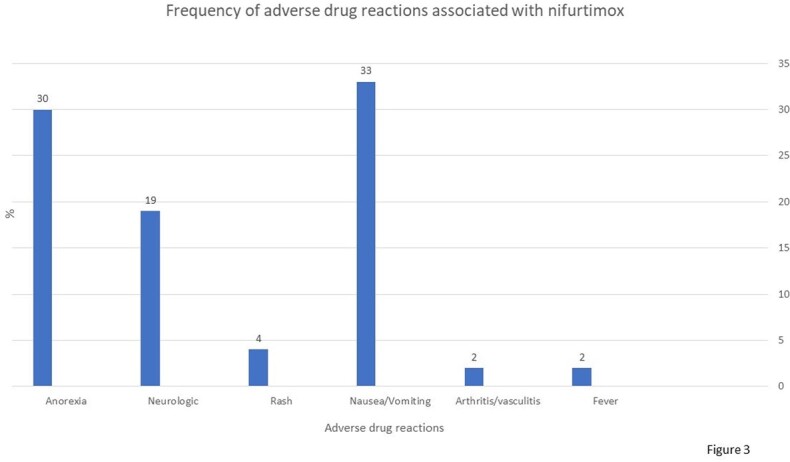

**Conclusion:**

ChD continues to be endemic in Chile. Screening in blood donors and pregnant women is the main approach to diagnosis. The importance of its detection in pregnancy is both, the prevention of vertical transmission and the opportunity for early curative treatment. The incidence of ADRs is lower than that described in other series; a small percentage of these cases led to the discontinuation of treatment.

**Disclosures:**

**All Authors**: No reported disclosures

